# 3,5-Dimethyl-1-(4-nitro­benz­yl)pyridinium bis­(benzene-1,2-dithiol­ato-κ^2^ *S*,*S*′)nickelate(III)

**DOI:** 10.1107/S1600536812009828

**Published:** 2012-03-14

**Authors:** Guang-Xiang Liu

**Affiliations:** a School of Biochemical and Environmental Engineering, Nanjing Xiaozhuang University, Nanjing 211171, People’s Republic of China

## Abstract

The asymmetric unit of the title compound, (C_14_H_15_N_2_O_2_)[Ni(C_6_H_4_S_2_)_2_], contains one cation and two halves of two centrosymmetric crystallographically independent anions. In the anions, the Ni^III^ atoms are coordinated by four S atoms in a distorted square-planar geometry. In the cation, the dihedral angle between the pyridine and benzene rings is 88.66 (17)°. In the crystal, anions and cations inter­act through C—H⋯S and C—H⋯O hydrogen bonds.

## Related literature

For general background to the properties and applications of metal complexes of 1,2-dithiol­ate ligands, see: Robertson & Cronin (2002 ▶); Kato (2004 ▶); Cassoux (1999 ▶); Canadell (1999 ▶); Akutagawa & Nakamura (2000 ▶); Ren *et al.* (2002 ▶, 2004 ▶, 2008 ▶). For a related structure, see: Liu *et al.* (2007 ▶).
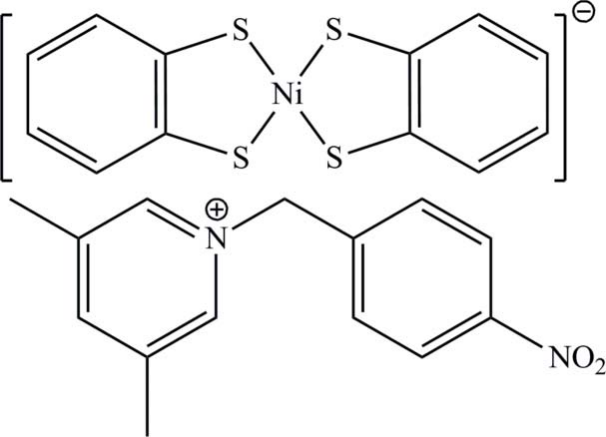


## Experimental

### 

#### Crystal data

(C_14_H_15_N_2_O_2_)[Ni(C_6_H_4_S_2_)_2_]
*M* *_r_* = 582.41
Triclinic, 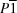

*a* = 7.6114 (14) Å
*b* = 12.010 (2) Å
*c* = 15.317 (3) Å
α = 84.546 (3)°
β = 85.927 (2)°
γ = 72.435 (3)°
*V* = 1327.5 (4) Å^3^
*Z* = 2
Mo *K*α radiation
μ = 1.07 mm^−1^
*T* = 293 K
0.12 × 0.10 × 0.04 mm

#### Data collection

Bruker SMART APEX CCD area-detector diffractometer
Absorption correction: multi-scan (*SADABS*; Bruker, 2000 ▶) *T*_min_ = 0.882, *T*_max_ = 0.958
6651 measured reflections
4578 independent reflections
2424 reflections with *I* > 2σ(*I*)
*R*_int_ = 0.033

#### Refinement

*R*[*F* ^2^ > 2σ(*F* ^2^)] = 0.048
*wR*(*F* ^2^) = 0.069
*S* = 0.95
4578 reflections
321 parameters
H-atom parameters constrained
Δρ_max_ = 0.23 e Å^−3^
Δρ_min_ = −0.18 e Å^−3^

### 

Data collection: *SMART* (Bruker, 2000 ▶); cell refinement: *SAINT* (Bruker, 2000 ▶); data reduction: *SAINT*; program(s) used to solve structure: *SHELXS97* (Sheldrick, 2008 ▶); program(s) used to refine structure: *SHELXL97* (Sheldrick, 2008 ▶); molecular graphics: *SHELXTL* (Sheldrick, 2008 ▶); software used to prepare material for publication: *SHELXTL*.

## Supplementary Material

Crystal structure: contains datablock(s) I, global. DOI: 10.1107/S1600536812009828/rz2716sup1.cif

Structure factors: contains datablock(s) I. DOI: 10.1107/S1600536812009828/rz2716Isup2.hkl

Additional supplementary materials:  crystallographic information; 3D view; checkCIF report

## Figures and Tables

**Table 1:** Hydrogen-bond geometry (Å, °)

*D*—H⋯*A*	*D*—H	H⋯*A*	*D*⋯*A*	*D*—H⋯*A*
C19—H19*A*⋯S2^i^	0.97	2.88	3.697 (4)	143
C22—H22⋯O1^ii^	0.93	2.57	3.484 (7)	167

Symmetry codes: (i) 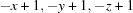; (ii) 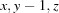.

**Fig. 1.**
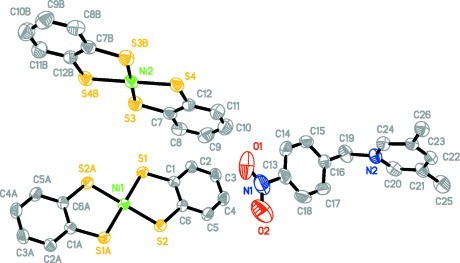
The asymmetric unit of the title complex, with displacement ellipsoids drawn at the 30% probability level. Hydrogen atoms are omitted for clarity. Atoms marked with suffixes A and B are related to those with no suffixes by the symmetry codes (1-x, 2-y, 1-z) and (1-x, 2-y, -z) respectively.

**Fig. 2.**
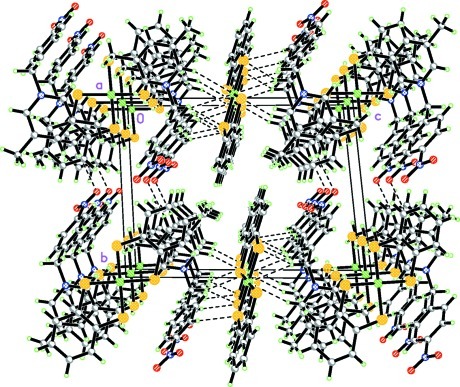
Packing diagram of the title complex viewed along the a axis. Hydrogen bonds are shown as dashed lines.

## supplementary crystallographic information

### Comment

Metal complexes of 1,2-dithiolate ligands have been intensively studied because of their novel properties and applications in the areas of molecular conducting, magnetic materials, nonlinear optics, and others (Robertson *et al.*, 2002; Kato, 2004). Over the last decade, a large number of new dithiolene ligands and resultant complexes have been prepared to optimize the molecular properties in an effort to prepare novel and advanced material, whose molecular arrangement can be sensitively affected by strong and directional noncovalent interactions (Cassoux, 1999; Canadell, 1999; Akutagawa & Nakamura, 2000). Although the closed-shell cations make no contribution to the conductivity and magnetism, their size and shapes play a predominant role in influencing the crystal structure and consequently, in altering the electronic and magnetic properties. Recently, using benzylpyridinium derivatives ([RBzPy]^+^) as the counter cation of [*M*(mnt)_2_]^-^ (*M* = Ni, Pd, and Pt; mnt^2–^ = maleonitriledithiolate), a series of ion-pair complexes with segregated columnar stacks of cations and anions have been reported (Ren *et al.*, 2002, 2008). The quasi-one-dimensional magnetic nature of these complexes was attributed to intermolecular π orbital interactions within the anionic columns. Furthermore, for some complexes, spin-Peierls-like transition was observed (Ren *et al.*, 2004). More presently, we are devoted to our research interesting on the molecular magnets self-assembled from [Ni(bdt)_2_]^-^ ion (bdt is benzene-1,2-dithiolato) due to its molecular and electronic structure resembling [Ni(mnt)_2_]^-^ ion, which is expected to obtain new series of molecular magnets with peculiar magnetic phase transition *via* incorporating the benzylpyridinium derivatives into the [Ni(bdt)_2_]^-^ spin system. We report herein the synthesis and crystal structure of the title compound, a new ion-pair complex.

As shown in Fig. 1, the asymmetric unit of the title complex contains two different, independent halves of centrosymmetric [Ni(bdt)_2_]^-^ anions and one [NO_2_BzPy(CH_3_)_2_]^+^ cation. The nickel atoms are each coordinated by four sulphur atoms in square-planar geometry. As for the Ni1-containing unit, the Ni1—S1 and Ni1—S2 distances are 2.1419 (11) and 2.1490 (10) Å, respectively. These values are in agreement with those reported for an analogous [Ni(bdt)_2_]^-^ complex (Liu *et al.*, 2007). The S—Ni—S bond angle within the five-member ring is 91.77 (4)°, which is slightly larger than that observed in the complex with substituent groups on benzene rings (Liu *et al.*, 2007). There exists a dihedral angle of 5.36 (6)° between the C_6_S_2_ and NiS_2_ planes, with atom Ni1 deviating 0.165 (5) Å from the C_6_S_2_ plane. In the Ni2-containing unit, the Ni—S bonds range 2.1425 (11) to 2.1474 (11) Å and the S—Ni—S bond angle within the five member ring is 91.56 (4)°, which is in agreement with that of the Ni1-containing unit. The Ni2 atom deviates by 0.017 Å from the C_6_S_2_ plane and the dihedral angle between the C_6_S_2_ and NiS_2_ planes is 1.12 (4)°. The independent C_6_S_2_ planes are nearly perpendicular to each other forming a dihedral angle of 78.33 (8)°. In the cation, the dihedral angles formed by the N2/C19/C16 reference plane are 65.68 (14)° for the phenyl ring and 48.66 (15)° for the pyridine ring, respectively. The phenyl ring and the pyridine ring make a dihedral angle of 88.66 (17)°. The packing of two anionic units differ from each other. The Ni2-containing units stack in face-to-face fashion with an alternating arrangement of the anions and cations. Conversely, the Ni1-containing units stack in side-by-side fashion in which the anions with uniform spaced arrangements form one-dimensional chains along the *a* axis. The shortest separation between adjacent Ni(III) ions is 7.611 (14)Å. In the crystal (Fig. 2), anions and cation are held together *via* C—H···O and C—H···S hydrogen bonding interactions (Table 1).

### Experimental

Under argon atmosphere at room temperature, benzene-1,2-dithiol (284 mg, 2 mmol) was added to a solution of sodium metal (92 mg, 4 mmol) in 25 ml of absolute methanol. A solution of NiCl_2_.6H_2_O (240 mg, 1 mmol) in methanol was added, resulting in the formation of a muddy red-brown color. Following this, 1-(4-nitrobenzyl)-3,5-dimethylpyridinium bromide (646 mg, 2 mmol) was added, and the mixture allowed to stand with stirring for 1 h and then stirred for 24 h in air. The colour of the mixture gradually turned green, indicating oxidation from a dianionic species to the more stable monoanionic form. The precipitate was washed with absolute methanol and ether and then dried. The crude product was recrystallized twice from methylene chloride to give black needles in ~61% yield.

### Refinement

H atoms were positioned geometrically, with C—H = 0.93, 0.97 and 0.96 Å for aromatic, methylene and methyl H atoms, respectively, and constrained to ride on their parent atoms, with *U*_iso_(H) = xU_eq_(C), where *x* = 1.5 for methyl H and 1.2 for all other H atoms.

### Crystal data

**Table:**

(C_14_H_15_N_2_O_2_)[Ni(C_6_H_4_S_2_)_2_]	*Z* = 2
*M**_r_* = 582.41	*F*(000) = 602
Triclinic, *P*1	*D*_x_ = 1.457 Mg m^−^^3^
Hall symbol: -P 1	Mo *K*α radiation, λ = 0.71073 Å
*a* = 7.6114 (14) Å	Cell parameters from 1160 reflections
*b* = 12.010 (2) Å	θ = 2.7–18.1°
*c* = 15.317 (3) Å	µ = 1.07 mm^−^^1^
α = 84.546 (3)°	*T* = 293 K
β = 85.927 (2)°	Platelet, dark green
γ = 72.435 (3)°	0.12 × 0.10 × 0.04 mm
*V* = 1327.5 (4) Å^3^	

### Data collection

**Table:**

Bruker SMART APEX CCD area-detector diffractometer	4578 independent reflections
Radiation source: sealed tube	2424 reflections with *I* > 2σ(*I*)
Graphite monochromator	*R*_int_ = 0.033
phi and ω scans	θ_max_ = 25.0°, θ_min_ = 1.8°
Absorption correction: multi-scan (*SADABS*; Bruker, 2000)	*h* = −9→7
*T*_min_ = 0.882, *T*_max_ = 0.958	*k* = −14→7
6651 measured reflections	*l* = −18→18

### Refinement

**Table:**

Refinement on *F*^2^	Primary atom site location: structure-invariant direct methods
Least-squares matrix: full	Secondary atom site location: difference Fourier map
*R*[*F*^2^ > 2σ(*F*^2^)] = 0.048	Hydrogen site location: inferred from neighbouring sites
*wR*(*F*^2^) = 0.069	H-atom parameters constrained
*S* = 0.95	*w* = 1/[σ^2^(*F*_o_^2^) + (0.0002*P*)^2^ + 0.0986*P*] where *P* = (*F*_o_^2^ + 2*F*_c_^2^)/3
4578 reflections	(Δ/σ)_max_ < 0.001
321 parameters	Δρ_max_ = 0.23 e Å^−^^3^
0 restraints	Δρ_min_ = −0.18 e Å^−^^3^

### Special details

**Table:**

Geometry. All e.s.d.'s (except the e.s.d. in the dihedral angle between two l.s. planes) are estimated using the full covariance matrix. The cell e.s.d.'s are taken into account individually in the estimation of e.s.d.'s in distances, angles and torsion angles; correlations between e.s.d.'s in cell parameters are only used when they are defined by crystal symmetry. An approximate (isotropic) treatment of cell e.s.d.'s is used for estimating e.s.d.'s involving l.s. planes.
Refinement. Refinement of *F*^2^ against ALL reflections. The weighted *R*-factor *wR* and goodness of fit *S* are based on *F*^2^, conventional *R*-factors *R* are based on *F*, with *F* set to zero for negative *F*^2^. The threshold expression of *F*^2^ > σ(*F*^2^) is used only for calculating *R*-factors(gt) *etc*. and is not relevant to the choice of reflections for refinement. *R*-factors based on *F*^2^ are statistically about twice as large as those based on *F*, and *R*- factors based on ALL data will be even larger.

### Fractional atomic coordinates and isotropic or equivalent isotropic displacement parameters (Å^2^)

**Table:**

	*x*	*y*	*z*	*U*_iso_*/*U*_eq_	
Ni1	0.5000	1.0000	0.5000	0.0634 (2)	
Ni2	0.5000	1.0000	0.0000	0.0804 (3)	
O1	1.0908 (9)	0.4666 (4)	0.1105 (4)	0.213 (3)	
O2	1.2794 (8)	0.3723 (4)	0.2098 (4)	0.209 (2)	
S1	0.25357 (13)	0.96184 (8)	0.47173 (6)	0.0790 (3)	
S2	0.62478 (13)	0.82242 (8)	0.55057 (6)	0.0743 (3)	
S3	0.37839 (15)	0.99427 (10)	0.13058 (7)	0.0960 (4)	
S4	0.56664 (15)	0.81374 (9)	−0.00239 (7)	0.0905 (4)	
N1	1.1382 (9)	0.3946 (5)	0.1687 (4)	0.144 (2)	
N2	0.7941 (4)	−0.0362 (3)	0.2355 (2)	0.0813 (10)	
C1	0.2877 (5)	0.8163 (3)	0.5090 (2)	0.0672 (10)	
C2	0.1519 (5)	0.7605 (4)	0.5056 (2)	0.0836 (12)	
H2	0.0409	0.8011	0.4801	0.100*	
C3	0.1795 (6)	0.6467 (4)	0.5392 (3)	0.0935 (13)	
H3	0.0870	0.6112	0.5375	0.112*	
C4	0.3455 (7)	0.5851 (4)	0.5755 (3)	0.0986 (14)	
H4	0.3648	0.5077	0.5975	0.118*	
C5	0.4811 (6)	0.6366 (4)	0.5795 (3)	0.0879 (13)	
H5	0.5914	0.5945	0.6050	0.105*	
C6	0.4556 (5)	0.7532 (3)	0.5454 (2)	0.0675 (10)	
C7	0.4178 (5)	0.8468 (4)	0.1626 (3)	0.0822 (12)	
C8	0.3665 (6)	0.8083 (5)	0.2483 (3)	0.1024 (15)	
H8	0.3128	0.8620	0.2895	0.123*	
C9	0.3973 (7)	0.6910 (6)	0.2692 (4)	0.1223 (18)	
H9	0.3657	0.6653	0.3256	0.147*	
C10	0.4743 (7)	0.6097 (5)	0.2084 (5)	0.1249 (19)	
H10	0.4906	0.5305	0.2236	0.150*	
C11	0.5267 (6)	0.6459 (5)	0.1255 (4)	0.1112 (16)	
H11	0.5796	0.5911	0.0849	0.133*	
C12	0.5005 (5)	0.7659 (4)	0.1020 (3)	0.0807 (12)	
C13	1.0226 (8)	0.3148 (5)	0.1929 (4)	0.0967 (15)	
C14	0.8642 (8)	0.3328 (4)	0.1516 (3)	0.1004 (15)	
H14	0.8271	0.3947	0.1090	0.120*	
C15	0.7588 (6)	0.2589 (5)	0.1734 (3)	0.0923 (14)	
H15	0.6499	0.2707	0.1449	0.111*	
C16	0.8114 (7)	0.1673 (4)	0.2367 (3)	0.0781 (12)	
C17	0.9698 (7)	0.1526 (4)	0.2783 (3)	0.0931 (14)	
H17	1.0069	0.0917	0.3216	0.112*	
C18	1.0762 (6)	0.2269 (5)	0.2570 (4)	0.1038 (17)	
H18	1.1836	0.2169	0.2862	0.125*	
C19	0.6967 (6)	0.0853 (4)	0.2592 (3)	0.1079 (15)	
H19A	0.6652	0.0838	0.3217	0.130*	
H19B	0.5827	0.1146	0.2285	0.130*	
C20	0.7956 (6)	−0.1279 (5)	0.2931 (3)	0.0917 (14)	
H20	0.7412	−0.1142	0.3490	0.110*	
C21	0.8745 (6)	−0.2407 (5)	0.2722 (3)	0.0872 (13)	
C22	0.9529 (5)	−0.2555 (4)	0.1886 (3)	0.0822 (12)	
H22	1.0075	−0.3312	0.1721	0.099*	
C23	0.9537 (5)	−0.1628 (4)	0.1284 (3)	0.0688 (11)	
C24	0.8703 (5)	−0.0528 (4)	0.1546 (3)	0.0772 (12)	
H24	0.8666	0.0118	0.1154	0.093*	
C25	0.8743 (7)	−0.3422 (4)	0.3383 (3)	0.1314 (18)	
H25A	0.8212	−0.3944	0.3135	0.197*	
H25B	0.8031	−0.3134	0.3901	0.197*	
H25C	0.9987	−0.3835	0.3534	0.197*	
C26	1.0373 (5)	−0.1798 (3)	0.0372 (2)	0.0886 (13)	
H26A	1.1462	−0.1546	0.0311	0.133*	
H26B	0.9499	−0.1345	−0.0044	0.133*	
H26C	1.0694	−0.2612	0.0266	0.133*	

### Atomic displacement parameters (Å^2^)

**Table:**

	*U*^11^	*U*^22^	*U*^33^	*U*^12^	*U*^13^	*U*^23^
Ni1	0.0585 (5)	0.0708 (5)	0.0592 (4)	−0.0198 (3)	0.0019 (3)	0.0014 (3)
Ni2	0.0710 (5)	0.0819 (6)	0.0895 (6)	−0.0180 (4)	−0.0068 (4)	−0.0256 (4)
O1	0.268 (7)	0.095 (4)	0.276 (7)	−0.063 (4)	0.030 (5)	−0.008 (3)
O2	0.198 (5)	0.229 (5)	0.252 (6)	−0.129 (4)	−0.013 (4)	−0.053 (4)
S1	0.0712 (7)	0.0745 (8)	0.0922 (8)	−0.0240 (5)	−0.0167 (6)	0.0066 (6)
S2	0.0610 (7)	0.0754 (7)	0.0832 (7)	−0.0198 (5)	−0.0022 (5)	0.0088 (5)
S3	0.0972 (9)	0.0931 (9)	0.0948 (9)	−0.0189 (7)	0.0016 (7)	−0.0278 (7)
S4	0.0866 (8)	0.0843 (9)	0.1006 (9)	−0.0183 (6)	−0.0063 (7)	−0.0294 (7)
N1	0.131 (5)	0.120 (6)	0.196 (7)	−0.046 (5)	−0.004 (5)	−0.058 (4)
N2	0.089 (3)	0.087 (3)	0.072 (3)	−0.030 (2)	0.019 (2)	−0.024 (2)
C1	0.068 (3)	0.073 (3)	0.063 (3)	−0.025 (2)	0.000 (2)	−0.006 (2)
C2	0.078 (3)	0.080 (3)	0.098 (3)	−0.030 (3)	−0.010 (2)	−0.005 (3)
C3	0.095 (4)	0.080 (4)	0.114 (4)	−0.040 (3)	0.000 (3)	−0.005 (3)
C4	0.100 (4)	0.075 (3)	0.125 (4)	−0.037 (3)	−0.004 (3)	0.007 (3)
C5	0.078 (3)	0.072 (3)	0.107 (3)	−0.016 (2)	−0.005 (3)	0.008 (3)
C6	0.068 (3)	0.066 (3)	0.068 (3)	−0.022 (2)	0.007 (2)	−0.005 (2)
C7	0.068 (3)	0.088 (4)	0.088 (3)	−0.014 (2)	−0.013 (3)	−0.020 (3)
C8	0.092 (4)	0.121 (5)	0.091 (4)	−0.023 (3)	−0.012 (3)	−0.015 (3)
C9	0.098 (4)	0.126 (5)	0.133 (5)	−0.027 (4)	−0.011 (3)	0.019 (5)
C10	0.121 (5)	0.104 (5)	0.143 (5)	−0.026 (3)	−0.017 (4)	0.002 (4)
C11	0.111 (4)	0.093 (4)	0.122 (5)	−0.019 (3)	−0.012 (3)	−0.008 (4)
C12	0.066 (3)	0.076 (3)	0.101 (4)	−0.019 (2)	−0.021 (3)	−0.004 (3)
C13	0.107 (5)	0.077 (4)	0.110 (4)	−0.024 (3)	0.000 (4)	−0.041 (3)
C14	0.115 (5)	0.064 (4)	0.108 (4)	−0.001 (3)	−0.018 (4)	−0.012 (3)
C15	0.081 (4)	0.091 (4)	0.097 (4)	−0.002 (3)	−0.025 (3)	−0.029 (3)
C16	0.081 (4)	0.076 (3)	0.072 (3)	−0.010 (3)	0.006 (3)	−0.027 (3)
C17	0.092 (4)	0.101 (4)	0.077 (3)	−0.012 (3)	−0.021 (3)	−0.006 (3)
C18	0.071 (4)	0.131 (5)	0.112 (4)	−0.022 (3)	−0.022 (3)	−0.040 (4)
C19	0.107 (4)	0.114 (4)	0.107 (4)	−0.034 (3)	0.037 (3)	−0.051 (3)
C20	0.100 (4)	0.124 (4)	0.068 (3)	−0.062 (3)	0.016 (3)	−0.010 (3)
C21	0.103 (4)	0.094 (4)	0.083 (4)	−0.057 (3)	0.002 (3)	−0.011 (3)
C22	0.088 (3)	0.081 (3)	0.084 (3)	−0.031 (2)	−0.006 (3)	−0.019 (3)
C23	0.059 (3)	0.084 (3)	0.062 (3)	−0.017 (2)	−0.001 (2)	−0.016 (3)
C24	0.080 (3)	0.087 (3)	0.062 (3)	−0.023 (2)	0.014 (2)	−0.012 (2)
C25	0.193 (5)	0.128 (4)	0.102 (4)	−0.101 (4)	0.002 (4)	0.019 (3)
C26	0.089 (3)	0.097 (3)	0.076 (3)	−0.021 (2)	0.012 (2)	−0.024 (2)

### Geometric parameters (Å, º)

**Table:**

Ni1—S1^i^	2.1419 (11)	C9—H9	0.9300
Ni1—S1	2.1419 (11)	C10—C11	1.373 (5)
Ni1—S2^i^	2.1490 (10)	C10—H10	0.9300
Ni1—S2	2.1490 (10)	C11—C12	1.408 (5)
Ni2—S4^ii^	2.1425 (11)	C11—H11	0.9300
Ni2—S4	2.1425 (11)	C13—C14	1.350 (6)
Ni2—S3	2.1474 (11)	C13—C18	1.359 (6)
Ni2—S3^ii^	2.1474 (11)	C14—C15	1.372 (5)
O1—N1	1.176 (6)	C14—H14	0.9300
O2—N1	1.230 (6)	C15—C16	1.377 (5)
S1—C1	1.733 (4)	C15—H15	0.9300
S2—C6	1.740 (4)	C16—C17	1.361 (5)
S3—C7	1.733 (4)	C16—C19	1.503 (5)
S4—C12	1.739 (4)	C17—C18	1.379 (5)
N1—C13	1.491 (7)	C17—H17	0.9300
N2—C24	1.336 (4)	C18—H18	0.9300
N2—C20	1.343 (4)	C19—H19A	0.9700
N2—C19	1.491 (4)	C19—H19B	0.9700
C1—C6	1.398 (4)	C20—C21	1.364 (5)
C1—C2	1.398 (5)	C20—H20	0.9300
C2—C3	1.373 (4)	C21—C22	1.378 (5)
C2—H2	0.9300	C21—C25	1.510 (5)
C3—C4	1.382 (5)	C22—C23	1.377 (4)
C3—H3	0.9300	C22—H22	0.9300
C4—C5	1.361 (5)	C23—C24	1.367 (4)
C4—H4	0.9300	C23—C26	1.499 (4)
C5—C6	1.408 (4)	C24—H24	0.9300
C5—H5	0.9300	C25—H25A	0.9600
C7—C12	1.386 (5)	C25—H25B	0.9600
C7—C8	1.418 (5)	C25—H25C	0.9600
C8—C9	1.366 (5)	C26—H26A	0.9600
C8—H8	0.9300	C26—H26B	0.9600
C9—C10	1.385 (6)	C26—H26C	0.9600
			
S1^i^—Ni1—S1	180.00 (5)	C7—C12—C11	119.3 (4)
S1^i^—Ni1—S2^i^	91.77 (4)	C7—C12—S4	119.8 (4)
S1—Ni1—S2^i^	88.23 (4)	C11—C12—S4	121.0 (4)
S1^i^—Ni1—S2	88.23 (4)	C14—C13—C18	121.1 (5)
S1—Ni1—S2	91.77 (4)	C14—C13—N1	119.2 (6)
S2^i^—Ni1—S2	180.00 (5)	C18—C13—N1	119.6 (6)
S4^ii^—Ni2—S4	180.00 (6)	C13—C14—C15	119.2 (5)
S4^ii^—Ni2—S3	88.44 (4)	C13—C14—H14	120.4
S4—Ni2—S3	91.56 (4)	C15—C14—H14	120.4
S4^ii^—Ni2—S3^ii^	91.56 (4)	C14—C15—C16	121.1 (5)
S4—Ni2—S3^ii^	88.44 (4)	C14—C15—H15	119.5
S3—Ni2—S3^ii^	180.00 (6)	C16—C15—H15	119.5
C1—S1—Ni1	105.34 (14)	C17—C16—C15	118.4 (5)
C6—S2—Ni1	104.83 (14)	C17—C16—C19	120.8 (5)
C7—S3—Ni2	105.32 (17)	C15—C16—C19	120.8 (5)
C12—S4—Ni2	104.68 (17)	C16—C17—C18	120.9 (5)
O1—N1—O2	127.5 (8)	C16—C17—H17	119.6
O1—N1—C13	117.4 (7)	C18—C17—H17	119.6
O2—N1—C13	114.9 (7)	C13—C18—C17	119.3 (5)
C24—N2—C20	120.6 (4)	C13—C18—H18	120.3
C24—N2—C19	119.1 (4)	C17—C18—H18	120.3
C20—N2—C19	120.1 (4)	N2—C19—C16	112.4 (3)
C6—C1—C2	118.8 (4)	N2—C19—H19A	109.1
C6—C1—S1	118.9 (3)	C16—C19—H19A	109.1
C2—C1—S1	122.3 (3)	N2—C19—H19B	109.1
C3—C2—C1	121.1 (4)	C16—C19—H19B	109.1
C3—C2—H2	119.5	H19A—C19—H19B	107.8
C1—C2—H2	119.5	N2—C20—C21	121.9 (4)
C2—C3—C4	119.8 (4)	N2—C20—H20	119.1
C2—C3—H3	120.1	C21—C20—H20	119.1
C4—C3—H3	120.1	C20—C21—C22	116.4 (4)
C5—C4—C3	120.7 (4)	C20—C21—C25	120.8 (5)
C5—C4—H4	119.7	C22—C21—C25	122.8 (5)
C3—C4—H4	119.7	C23—C22—C21	122.8 (4)
C4—C5—C6	120.4 (4)	C23—C22—H22	118.6
C4—C5—H5	119.8	C21—C22—H22	118.6
C6—C5—H5	119.8	C24—C23—C22	116.9 (4)
C1—C6—C5	119.2 (4)	C24—C23—C26	120.6 (4)
C1—C6—S2	119.1 (3)	C22—C23—C26	122.4 (4)
C5—C6—S2	121.7 (3)	N2—C24—C23	121.4 (4)
C12—C7—C8	120.1 (4)	N2—C24—H24	119.3
C12—C7—S3	118.4 (4)	C23—C24—H24	119.3
C8—C7—S3	121.5 (4)	C21—C25—H25A	109.5
C9—C8—C7	118.9 (5)	C21—C25—H25B	109.5
C9—C8—H8	120.5	H25A—C25—H25B	109.5
C7—C8—H8	120.5	C21—C25—H25C	109.5
C8—C9—C10	121.5 (6)	H25A—C25—H25C	109.5
C8—C9—H9	119.2	H25B—C25—H25C	109.5
C10—C9—H9	119.2	C23—C26—H26A	109.5
C11—C10—C9	120.0 (6)	C23—C26—H26B	109.5
C11—C10—H10	120.0	H26A—C26—H26B	109.5
C9—C10—H10	120.0	C23—C26—H26C	109.5
C10—C11—C12	120.2 (5)	H26A—C26—H26C	109.5
C10—C11—H11	119.9	H26B—C26—H26C	109.5
C12—C11—H11	119.9		
			
S1^i^—Ni1—S1—C1	68 (100)	C8—C7—C12—S4	−178.9 (3)
S2^i^—Ni1—S1—C1	−177.31 (12)	S3—C7—C12—S4	1.5 (4)
S2—Ni1—S1—C1	2.69 (12)	C10—C11—C12—C7	−1.4 (6)
S1^i^—Ni1—S2—C6	176.97 (12)	C10—C11—C12—S4	179.7 (4)
S1—Ni1—S2—C6	−3.03 (12)	Ni2—S4—C12—C7	2.2 (3)
S2^i^—Ni1—S2—C6	−59 (100)	Ni2—S4—C12—C11	−178.9 (3)
S4^ii^—Ni2—S3—C7	−175.48 (13)	O1—N1—C13—C14	−4.3 (9)
S4—Ni2—S3—C7	4.52 (13)	O2—N1—C13—C14	179.6 (5)
S3^ii^—Ni2—S3—C7	21 (100)	O1—N1—C13—C18	177.0 (6)
S4^ii^—Ni2—S4—C12	168 (100)	O2—N1—C13—C18	0.9 (8)
S3—Ni2—S4—C12	−3.86 (14)	C18—C13—C14—C15	−1.8 (7)
S3^ii^—Ni2—S4—C12	176.14 (14)	N1—C13—C14—C15	179.5 (4)
Ni1—S1—C1—C6	−1.6 (3)	C13—C14—C15—C16	0.3 (7)
Ni1—S1—C1—C2	177.0 (3)	C14—C15—C16—C17	0.9 (6)
C6—C1—C2—C3	1.7 (6)	C14—C15—C16—C19	−179.0 (4)
S1—C1—C2—C3	−177.0 (3)	C15—C16—C17—C18	−0.6 (6)
C1—C2—C3—C4	−1.3 (6)	C19—C16—C17—C18	179.3 (4)
C2—C3—C4—C5	1.0 (7)	C14—C13—C18—C17	2.1 (7)
C3—C4—C5—C6	−1.0 (7)	N1—C13—C18—C17	−179.2 (4)
C2—C1—C6—C5	−1.6 (5)	C16—C17—C18—C13	−0.8 (7)
S1—C1—C6—C5	177.1 (3)	C24—N2—C19—C16	−50.5 (5)
C2—C1—C6—S2	−179.6 (3)	C20—N2—C19—C16	133.2 (4)
S1—C1—C6—S2	−0.9 (4)	C17—C16—C19—N2	−65.6 (5)
C4—C5—C6—C1	1.3 (6)	C15—C16—C19—N2	114.3 (4)
C4—C5—C6—S2	179.2 (3)	C24—N2—C20—C21	0.2 (6)
Ni1—S2—C6—C1	2.9 (3)	C19—N2—C20—C21	176.4 (4)
Ni1—S2—C6—C5	−175.0 (3)	N2—C20—C21—C22	0.0 (6)
Ni2—S3—C7—C12	−4.4 (3)	N2—C20—C21—C25	180.0 (4)
Ni2—S3—C7—C8	176.0 (3)	C20—C21—C22—C23	0.2 (6)
C12—C7—C8—C9	−1.0 (6)	C25—C21—C22—C23	−179.7 (4)
S3—C7—C8—C9	178.6 (3)	C21—C22—C23—C24	−0.7 (6)
C7—C8—C9—C10	−1.1 (7)	C21—C22—C23—C26	−179.3 (4)
C8—C9—C10—C11	1.9 (8)	C20—N2—C24—C23	−0.8 (6)
C9—C10—C11—C12	−0.7 (8)	C19—N2—C24—C23	−177.0 (3)
C8—C7—C12—C11	2.2 (6)	C22—C23—C24—N2	1.0 (5)
S3—C7—C12—C11	−177.4 (3)	C26—C23—C24—N2	179.5 (3)

Symmetry codes: (i) −*x*+1, −*y*+2, −*z*+1; (ii) −*x*+1, −*y*+2, −*z*.

### Hydrogen-bond geometry (Å, º)

**Table:**

*D*—H···*A*	*D*—H	H···*A*	*D*···*A*	*D*—H···*A*
C19—H19*A*···S2^iii^	0.97	2.88	3.697 (4)	143
C22—H22···O1^iv^	0.93	2.57	3.484 (7)	167

Symmetry codes: (iii) −*x*+1, −*y*+1, −*z*+1; (iv) *x*, *y*−1, *z*.
